# Supplementary figures and images for: Fingolimod inhibits multiple stages of the HIV-1 life cycle
Source: PLoS Pathog. 2020 Aug 13;16(8):e1008679. doi: 10.1371/journal.ppat.1008679 (PMC7425850; doi:10.1371/journal.ppat.1008679)

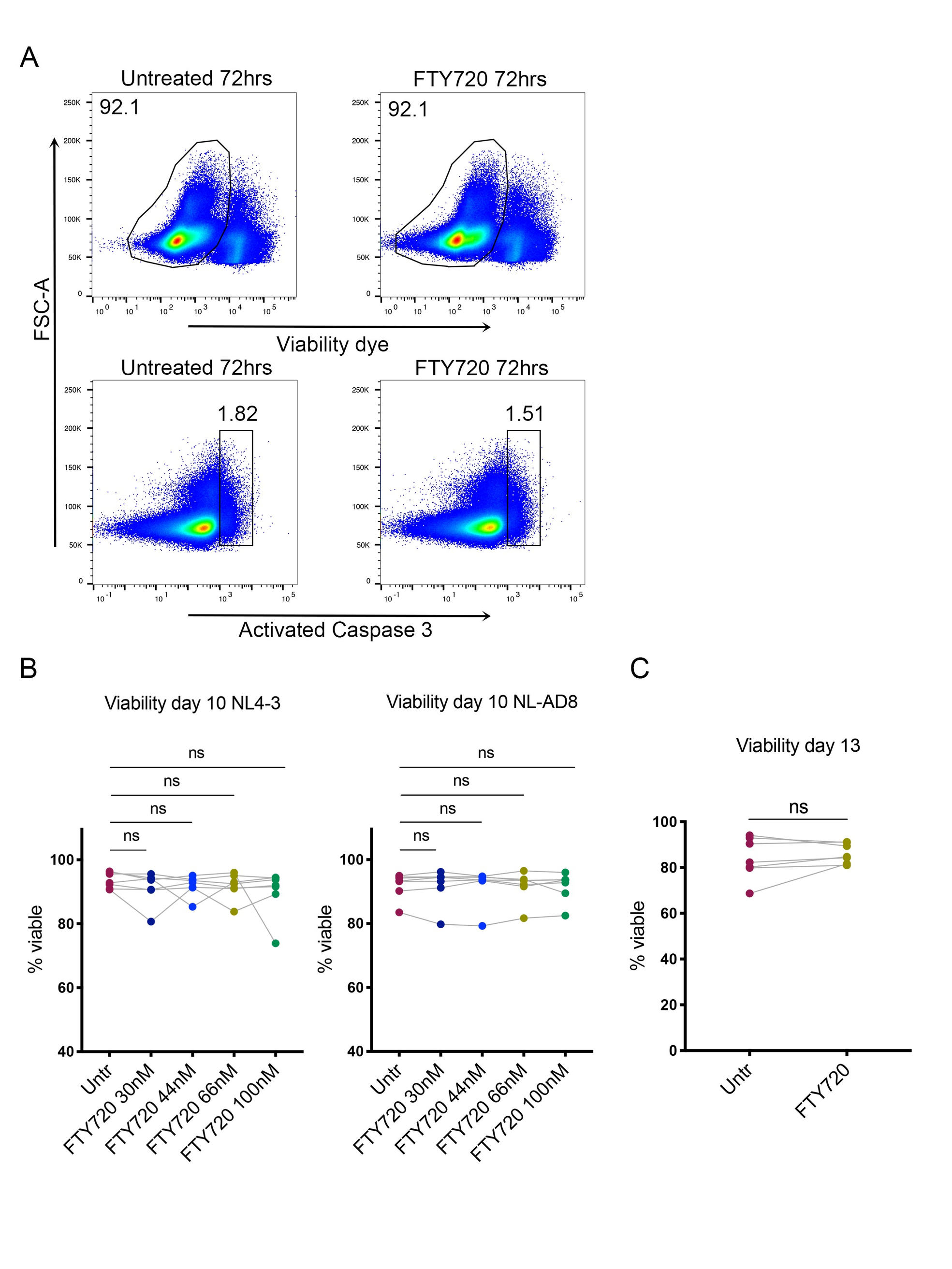

Supplement: S1 Fig — TCM obtained by immunomagnetic isolation from PBMCs were cultured with 66nM FTY720 for 72hrs and viability was evaluated by fixable viability dye and Activated Caspase 3 staining. Representative donor staining of viability dye and activated Caspase 3 on TCM. B-C. TCM obtained from naïve cells expanded in our primary cell model of latency were infected with NL4-3 or NL-AD8 and pre-treated +/-FTY720 (30–100 nM) and stained for flow cytometric assessment of viability at day 10 or 13. B. Viability of 7 donors infected with NL4-3 and 7 donors infected with NL-AD8 pre-treated +/-30-100nM FTY720 from day 5–7 and infected from day 7–10 (as in Fig 1A left schematic). C. Viability of 7 total donors infected with NL4-3 at day 7 (as in Fig 1A right schematic) and treated+/- 66nM FTY720 from day 10–13. For B-C., Wilcoxon signed-rank matched-paired tests were used for all comparisons. (TIF) [file ppat.1008679.s001.tif]

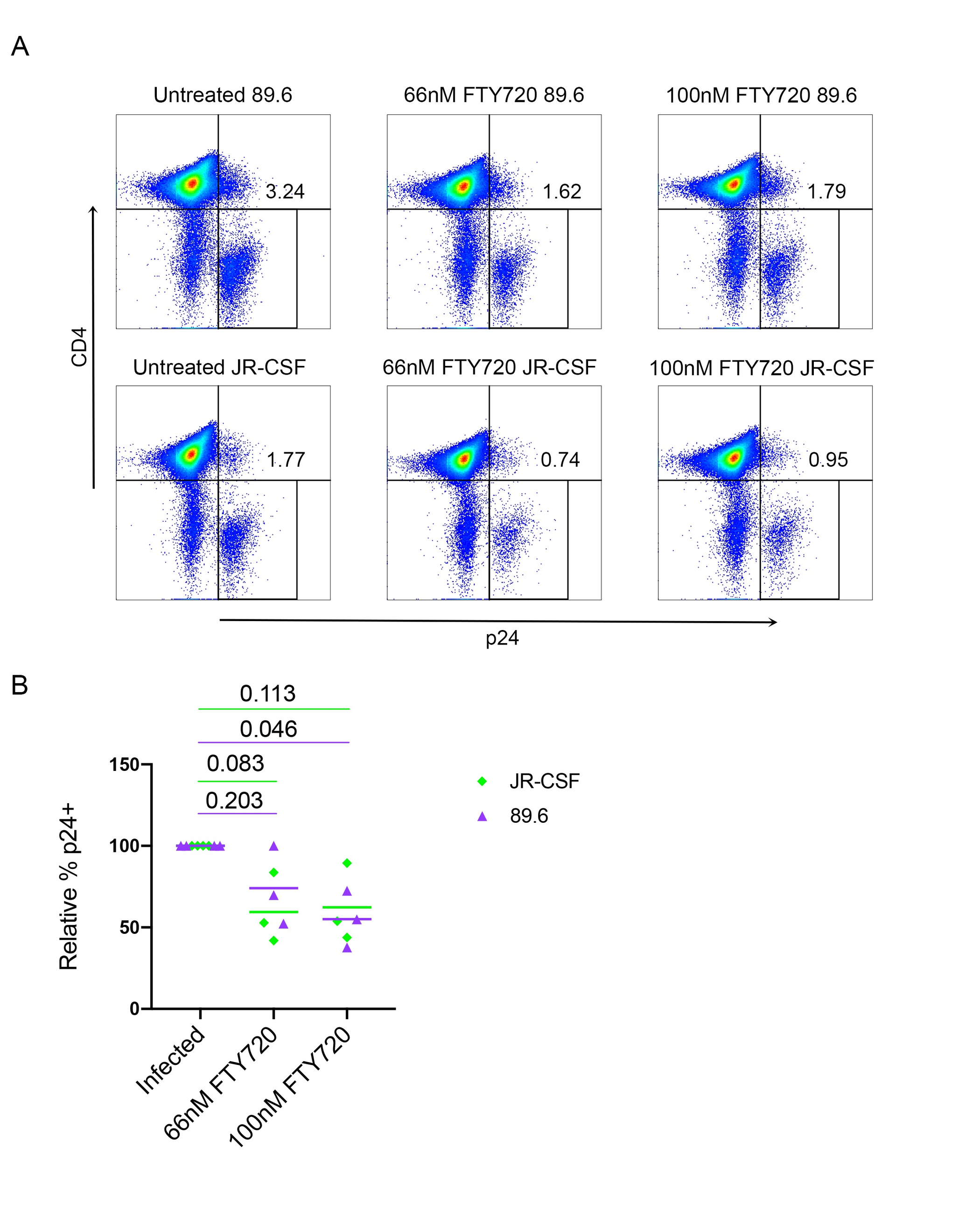

Supplement: S2 Fig — Primary CD4 T cells expanded and pre-treated at day 5 with 66 or 100nM FTY720 (as in Fig 1A, left schematic) were infected with dual-tropic HIV-1 (89.6) or R5-tropic HIV-1 (JR-CSF) at day 7 of culture. Frequency of p24+ cells was assessed at day 10 by flow cytometry (N = 3 donors for each virus and each concentration of FTY720). A. Representative donor infected with 89.6 and JR-CSF, either untreated or treated with two concentrations of FTY720. B. Summary of infections with 89.6 and JR-CSF. Data are expressed as the percent of infection in the FTY720-treated conditions relative to untreated. Mean + SD are shown; statistical comparison was performed by paired T-test and is color coded for each virus (green = JR-CSF, purple = 89.6). (TIF) [file ppat.1008679.s002.tif]

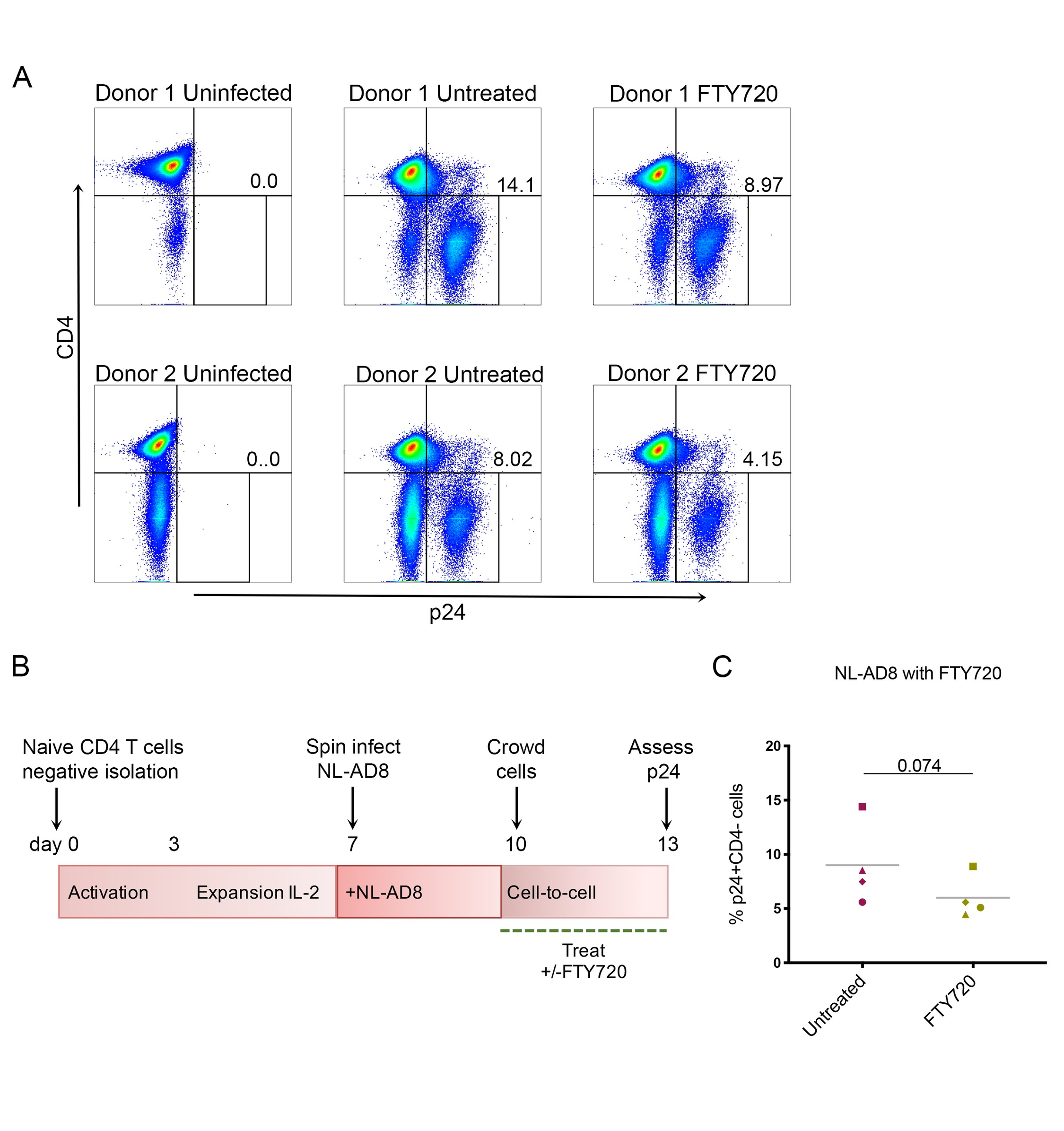

Supplement: S3 Fig — CD4 T cells were infected at day 7 with NL-AD8, crowded and treated with 66nM FTY720 from day 10–13, and assessed for frequency of infected cells by flow cytometry. A. Two representative donors from productive infection (day 13); uninfected, NL-AD8 infected (no treatment), and NL-AD8 infected (66nM FTY720 from day 10–13). B. Schematic of the experimental design. C. %p24+ cells at day 13 following treatment during crowding from day 10–13 with (or without) 66nM FTY720. Data comprise four total donors, each represented by a unique symbol. Statistical comparison was performed by paired T-test. (TIF) [file ppat.1008679.s003.tif]

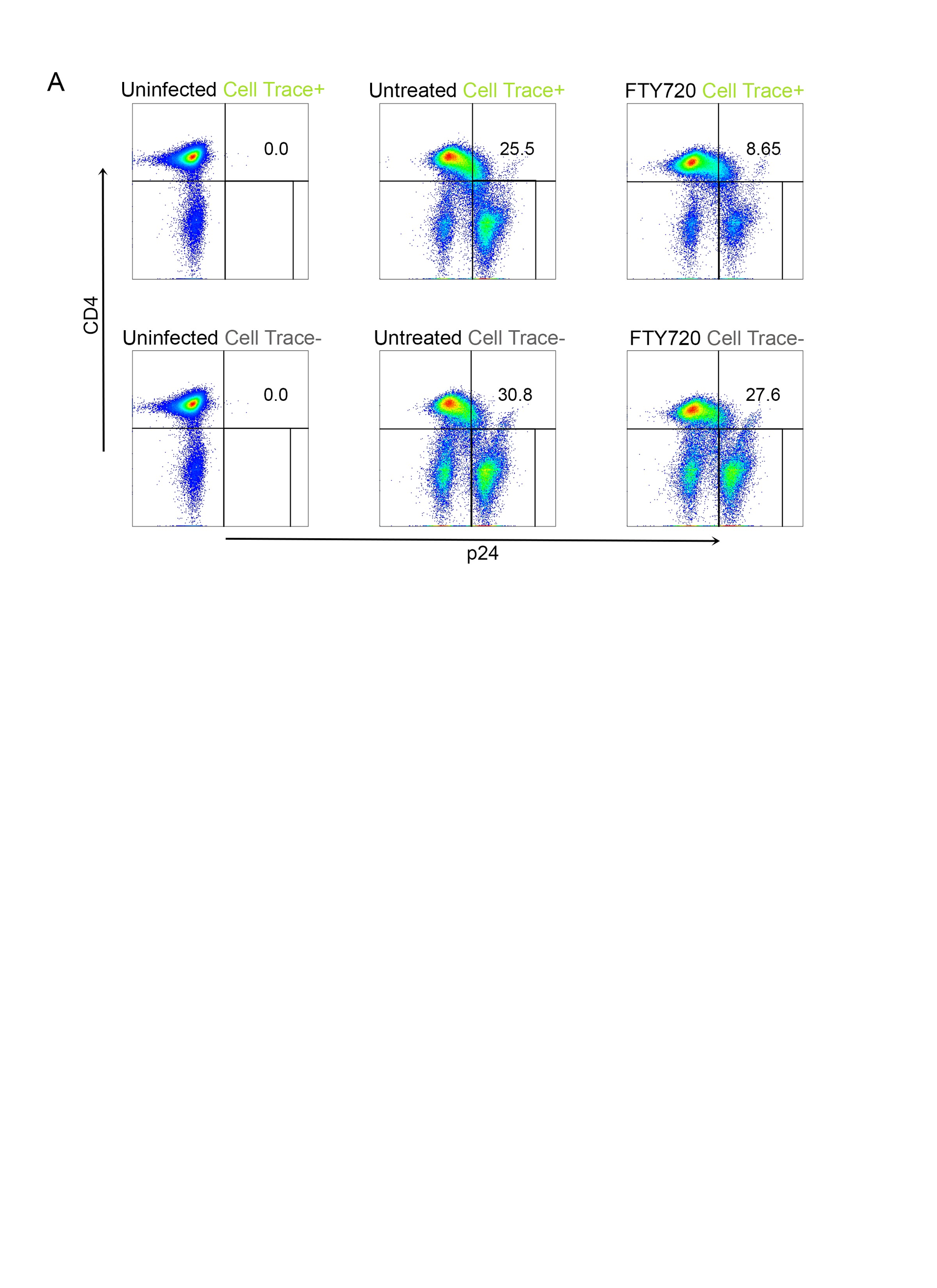

Supplement: S4 Fig — TCM either treated or untreated with 66nM FTY720 for 48 hrs were labeled with Cell Trace Yellow dye and co-cultured with unlabeled pre-crowded NL4-3 infected (producer) TCM. 48hrs later, pre-treated and untreated target cells were evaluated for intracellular expression of p24 by flow cytometry, gating on Cell Trace Yellow+ cells. Shown is one representative donor of two individual donors (uninfected, untreated, and FTY720 pre-treated Cell Trace-labeled target cells and unlabeled producer cells.) (TIF) [file ppat.1008679.s004.tif]

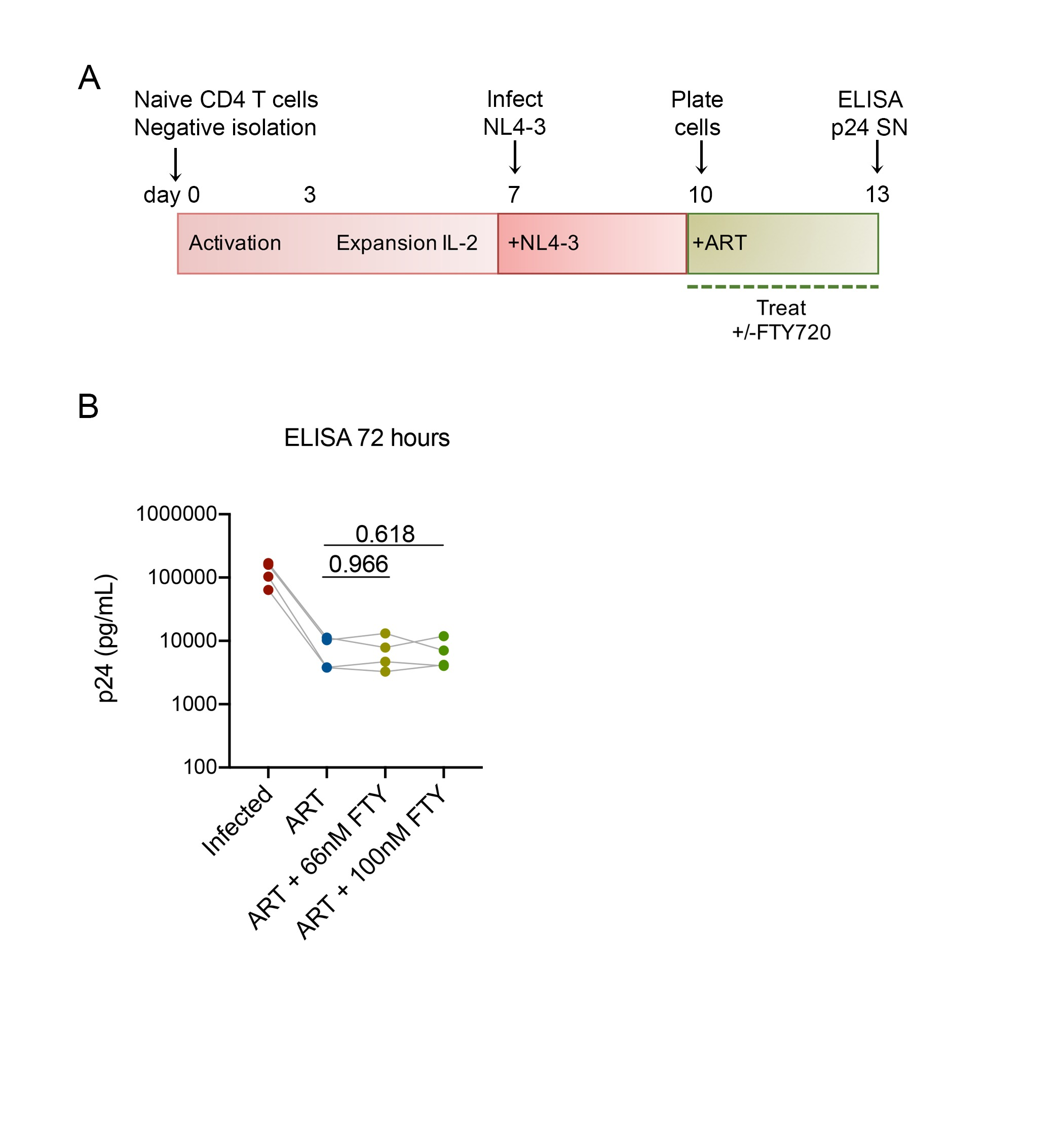

Supplement: S5 Fig — Primary CD4 T cells from our model were infected at day 7 and treated at day 10 with ART+/- 66 or 100nM FTY720, followed by assessment of p24-gag by ELISA at day 13 in order to determine the effect of FTY720 on viral release. A. Schematic of p24 ELISA following ART+/-FTY720 for 72 hours (days 10–13). B. Summary of p24 ELISA at day 13 following treatment of infected cells from day 10–13 with ART+/-FTY720 (either untreated or +FTY720, n = 4, statistical comparisons: paired T-test). (TIF) [file ppat.1008679.s005.tif]

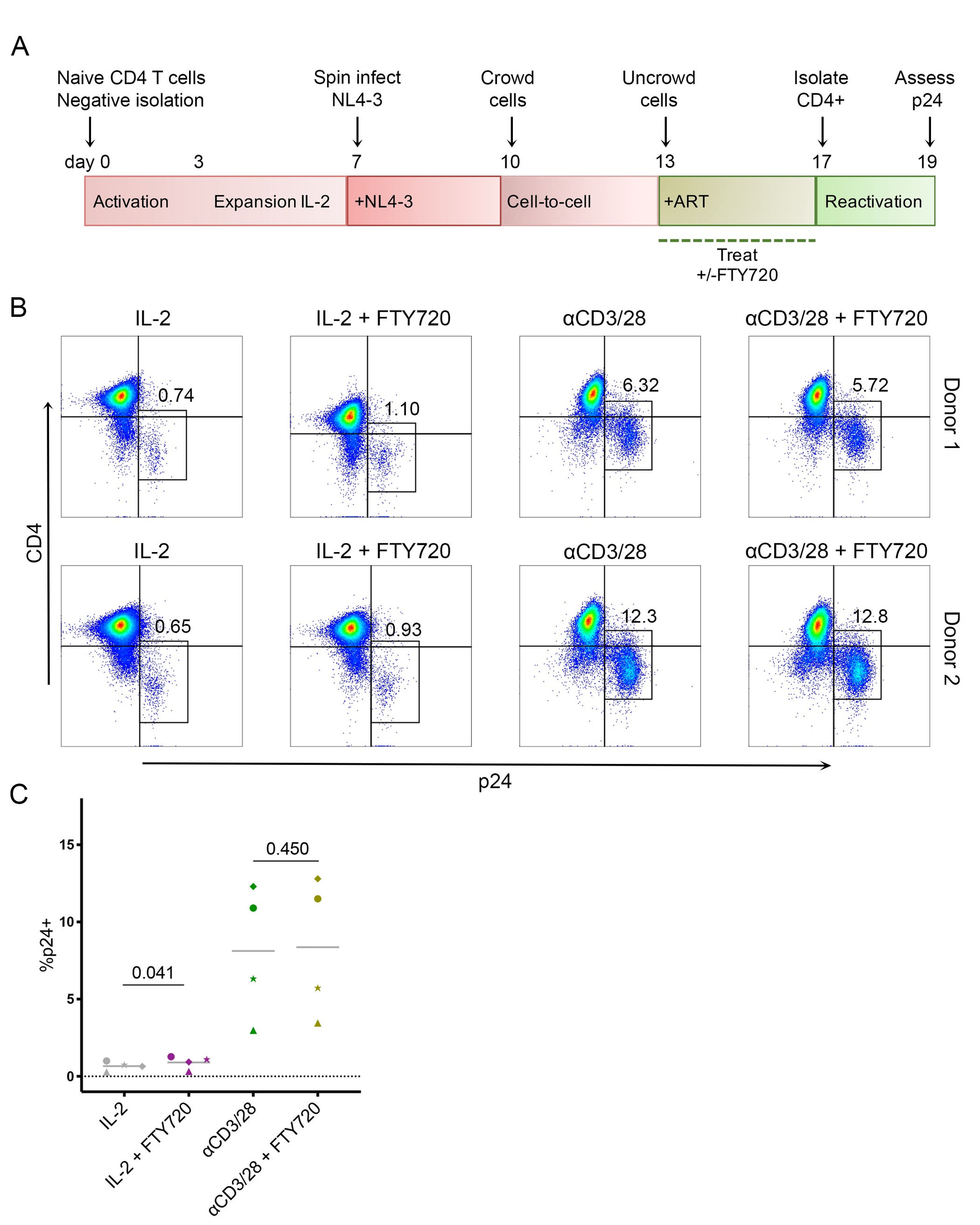

Supplement: S6 Fig — Primary CD4 T cells from our model of HIV latency were infected with NL4-3 at day 7, crowded at day 10, uncrowded at day 13 and treated for 4 days with ART (1μM Raltegravir/ 0.5 μM Nelfinavir) in the presence or absence of 66nM FTY720 prior to isolation of non-productively infected (CD4+) cells at day 17 and reactivation of latent HIV-1 for 48 hours with αCD3/28 or IL-2 only control. Frequency of reactivated virus (%p24+ cells) was assessed by flow cytometry at day 19. A. Schematic of latency reversal following FTY720 treatment during ART. B. Two representative donors (of 4 individual donors) from day 19, following 48 hours of reactivation with αCD3/28 or IL-2 only control, either untreated or +66nM FTY720 from day 13–17. C. Summary of day 19 reactivation with αCD3/28 or IL-2 only control (either untreated or +66nM FTY720 from day 13–17, n = 4, both statistical comparisons: paired T-test). Mean is indicated and each donor is represented by a unique symbol. (TIF) [file ppat.1008679.s006.tif]

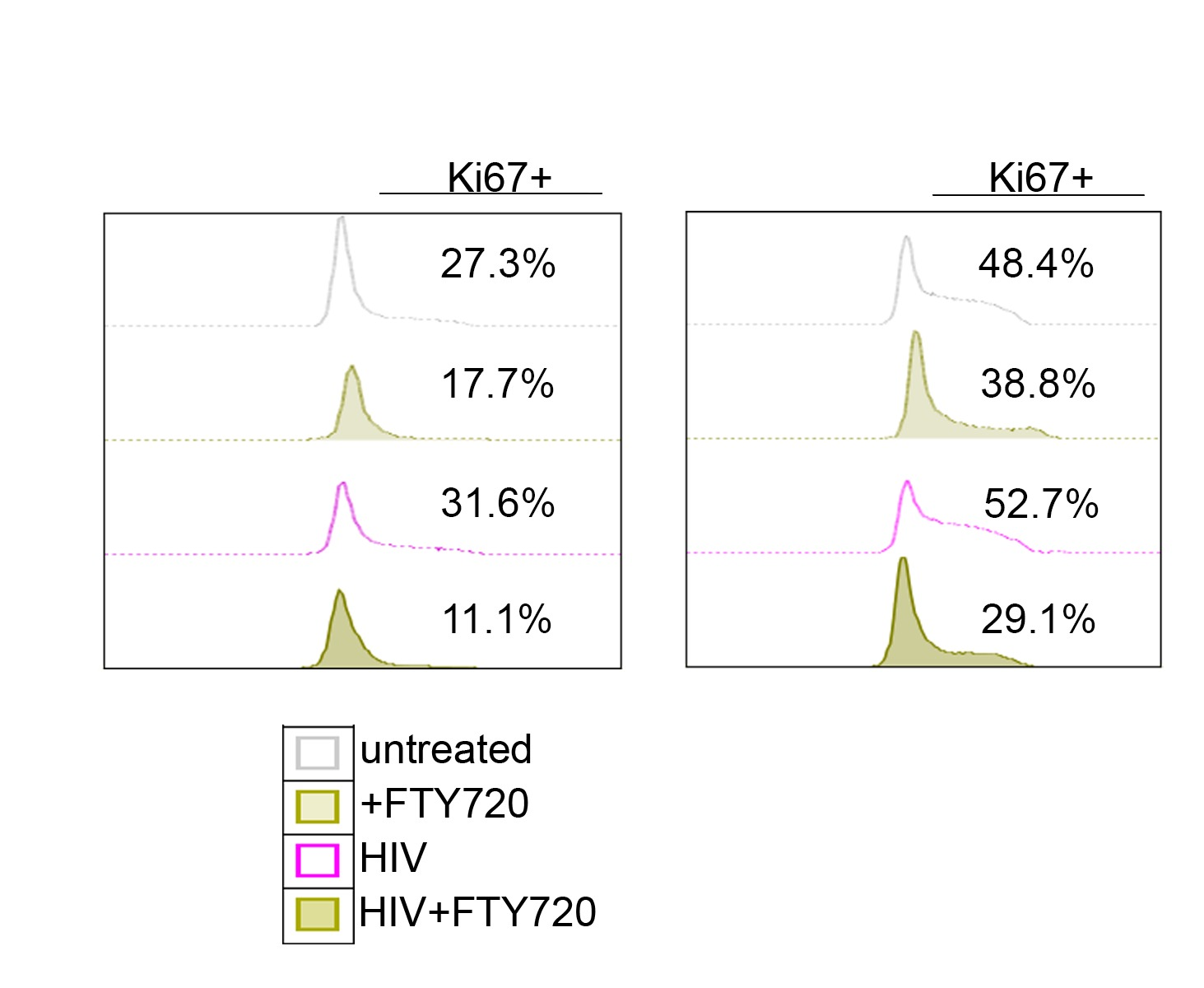

Supplement: S7 Fig — Primary CD4 T cells were cultured and expanded, infected with NL4-3 at day 7, crowded at day 10 and treated or not treated with 66nM FTY720, and stained at day 13 for flow cytometry to assess the expression of the proliferation marker Ki67. Two infected donors were stained. Grey dotted line: uninfected/ untreated; green dotted line with light fill: uninfected + 66nM FTY720; pink dotted line: NL4-3 infected/ untreated; dark green filled histogram: NL4-3 infected + 66nM FTY720. (TIF) [file ppat.1008679.s007.tif]

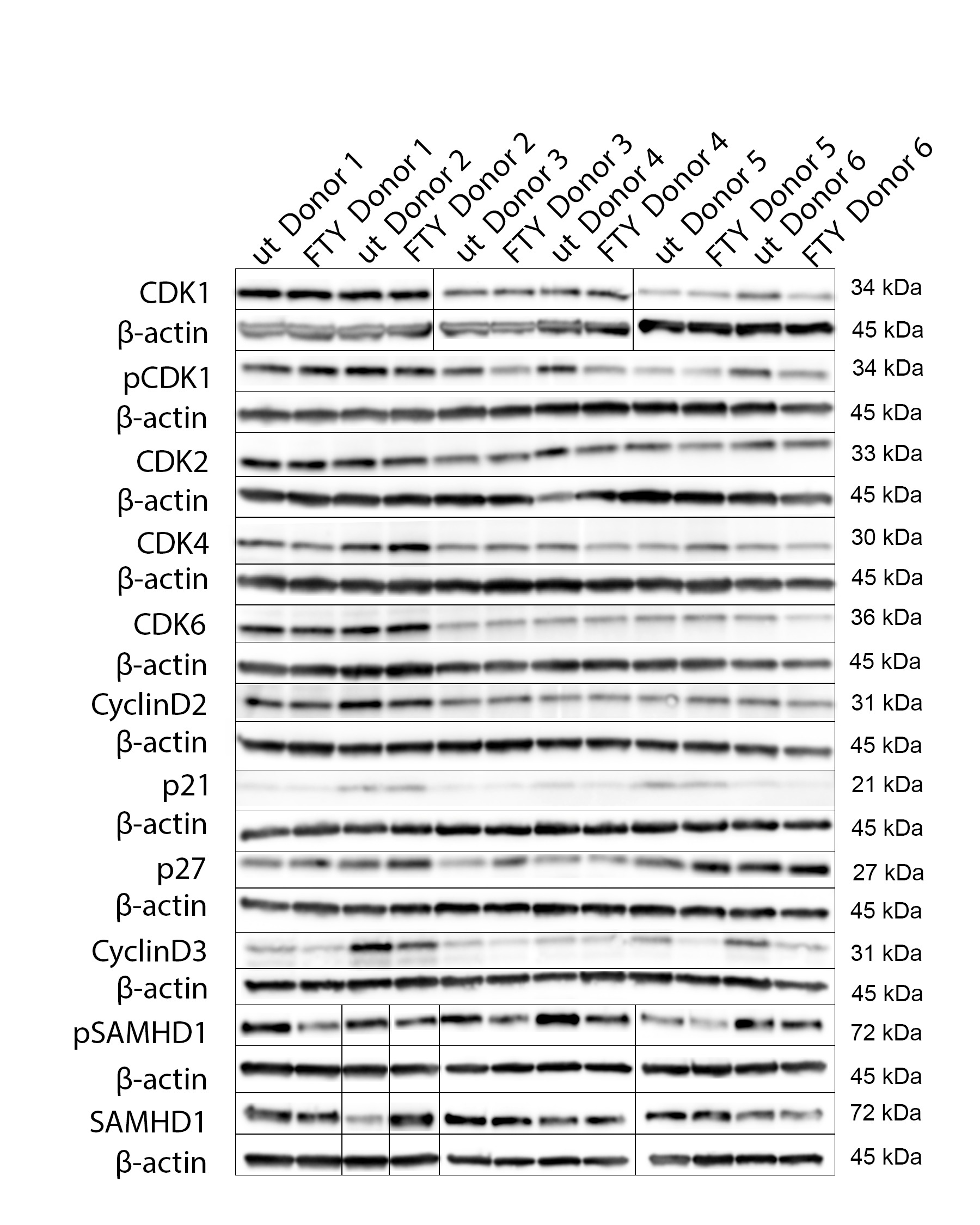

Supplement: S8 Fig — Primary CD4 T cells (Cultured T Central Memory cells) were treated for 24 hours +/- 100nM FTY720 and were lysed for Western Blot determination of protein levels of multiple Cyclin-Dependent kinases and Cyclins. Shown are 6 total donors assayed for: CDK1, pCDK1, CDK2, CDK4, CDK6, Cyclin D2, p21, p27, Cyclin D3, pSAMHD1 and total SAMHD1, with the molecular weight of each protein indicated. (TIF) [file ppat.1008679.s008.tif]
