# Supplementary material for: Fingolimod inhibits multiple stages of the HIV-1 life cycle
Source: PLoS Pathog. 2020 Aug 13;16(8):e1008679. doi: 10.1371/journal.ppat.1008679 (PMC7425850; doi:10.1371/journal.ppat.1008679)
Supplement: Attachment — Submitted filename: Response to Reviewers FTY720 FINAL.docx [file ppat.1008679.s009.docx]

We would like to thank each of the reviewers and the editor for taking the time to carefully evaluate our submitted manuscript and provide detailed feedback. We have performed several additional experiments which have significantly strengthened the manuscript, in addition to the increased clarity provided by modification to figures and analysis/ presentation of data suggested by reviewers. These additions and clarifications have enhanced the scientific rigor, readability, and strength of the manuscript and have clarified our conclusions. Please find below our responses to the suggestions of each reviewer (Reviewer comments are in green and our responses are in black text.) Thank you again for your time and effort.

**Reviewer #1:**

To validate their data, the authors should perform experiments in other models of HIV infection, including either cell lines or additional viral strains.

We would like to thank the reviewer for the valuable suggestion of repeated testing of our observations in additional models in order to add rigor to our observations. In addition to examining the effect of FTY720 on cell-free transmission and viability in detail with an X4-tropic HIV-1 molecular clone (NL4-3) and an R5-tropic HIV-1 molecular clone (NL-AD8, presented in **Figure 1** **B-E**) we have repeated our cell-free transmission experiments with two additional HIV strains, JR-CSF (R5-tropic patient isolate) and 89.6 (dual-tropic HIV-1 molecular clone), and the effect of inhibition of infection by FTY720 was again observed (presented in **Supplemental Figure 2** (**S2 Fig**)). The suggestion to consider utilization of cell lines is well-taken, and in future work we would like to optimize the use of a cell line for work with S1P and S1PRs. However, at this point, to our knowledge, a cell line does not exist that accurately represents the nuances of S1P signaling in primary human CD4 T cells. In previous work, we have utilized Jurkat cells as a positive control for S1P receptors 1-5 in RT-qPCR experiments as they abundantly expressed the mRNA for all S1P receptors (Resop et al, JACI, 2016); however, we and others have shown that in peripheral CD4 T cells the majority of the S1P receptors, with the exception of S1PR1 and S1PR4, may not be expressed at the mRNA level (Bosque et al, Cell Reports, 2017, figure below; Xiong et al, Sci Immunol, 2019). Moreover, in primary human CD4 lymphocytes S1P receptors are not detectable on the cell surface by flow cytometry (Mudd et al, Blood, 2014) as they are only transiently expressed in response to S1P gradients (Matloubian et al, Nature, 2004, Schwab et al, Science, 2005), further complicating the ability of cell lines to reproduce their signaling dynamics. Others have attempted to use cell lines to study S1P signaling but needed to transduce the cell lines with S1P receptors (Duquenne et al, AIDS, 2017). Therefore, to our knowledge, no cell line yet exists that closely represents S1P signaling dynamics in primary cells, although we would welcome the use of such a tool in the future.

Levels of S1PR in primary CD4 T cells by RNASeq

**Reviewer #2**:

Main concerns

1) Figure 1: is there a control for the activity of FTY720 on SP1R? Which SP1R are important for the effects observed? At least, the authors should discuss which SP1R are expressed on CD4 T cells and how HIV-1 infection modulates their expression.

We would like to thank the reviewer for bringing up this excellent point and for encouraging the inclusion of appropriate background information in the manuscript. We have expanded on the background regarding the expression of S1P receptors on CD4 T cells and how HIV infection may modulate their expression.

While the expression and myriad of functions of S1P receptors on primary CD4 T cells has yet to be fully elucidated, based on the literature it appears that S1P receptor 1 (S1PR1) is the main S1P receptor expressed, with S1PR4 also expressed. Early work in murine models (Matloubian et al, Nature 2004) and our work with primary human cells (Resop et al, JACI, 2016) demonstrated the relative abundance of S1PR1 on mature naïve CD4 T cells and the requirement of this receptor for thymic egress. However, flow cytometric staining for the receptor on peripheral T cells has proven challenging since it is upregulated prior to thymic egress and internalized on peripheral cells/ upon contact with Sphingosine-1-phosphate ((S1P), Matloubian et al, Nature, 2004, Resop et al, JACI, 2016). RNA Sequencing data from our lab (Bosque et al, Cell Reports, 2017) indicates that in Cultured T Central Memory Cells (T_CM_) from our model of HIV, S1PR1 and S1PR4 are the S1P receptors transcriptionally expressed (figure above). A recent publication by Xiong et al (Sci Immunol, 2019) confirmed the expression of S1PR1 and S1PR4 on various subsets of CD4 T cells, including T_CM_.

FTY720 has activity at four of the five S1P receptors (S1PR1, 3, 4 and 5; Brinkman et al, J Biol Chem, 2002) and has been shown to cause downregulation of S1PR1 in lymphocytes (Matloubian et al, Nature, 2004; Chiba et al, Pharmacol Ther, 2005). FTY720 acts as a “super-antagonist” on S1PR1, resulting in activation of the receptor followed by internalization and degradation (Grayler et al, FASEB J, 2004, Oo et al, J Biol Chem, 2007). Taken in the context of the established expression of S1PR1 on CD4 T cells and RNA Sequencing data from our model, this indicates that S1PR1 is likely the main receptor involved in our observed effects and is likely activated and internalized upon binding of FTY720. In clinical studies in the context of Multiple Sclerosis, FTY720 acted on T_CM_ to selectively reduce their presence in the periphery and retain them in the lymph node (Mehling et al, Neurology, 2008), but the mechanism of action was not fully elucidated. Specific antagonists of individual S1P receptors have been used to study S1PR1 and the other S1PR (Duquenne et al, AIDS, 2017, Resop et al, JACI, 2016); these specific antagonists could be employed to confirm the importance of each individual S1P receptor (S1PR1 vs S1PR4) for the activity of FTY720 in our model.

There is a paucity of literature on the effects of HIV-1 infection on modulation of S1P receptors on CD4 T cells; thus, we were initially drawn to ask the question of how S1P signaling may be involved in the progression of HIV infection. The Lederman group demonstrated that CD4 T cells from lymph nodes of chronically HIV-infected individuals had an impaired response to S1P when stimulated *ex-vivo* with the ligand. However, the group did not detect S1PR1 by flow cytometry on these cells but rather assessed functional response to S1P (Mudd et al, Blood, 2104). Recently, Duquenne et al (AIDS, 2017) demonstrated that S1PR1 is co-expressed with CCR5 on CD4 T cells and utilized an S1PR1 specific antagonist which resulted in reactivation of latent HIV; yet, they did not describe how HIV infection may have modulated expression of the receptor, which would be important knowing that it is co-expressed with the HIV-1 co-receptor CCR5. Additionally, Pino et al (PLoS Pathogens, 2019) reported that FTY720 retained the cytotoxic CD8 subset in lymph nodes of Rhesus Macaques and reduced viral load in T follicular helper cells; however, there was no evaluation of S1P receptor expression changes on CD4 T cells.

Importantly, especially during acute HIV infection uncontrolled by ART, there may be increased activation of remaining CD4 T cells which may result in an increase in CD69 expression. CD69 has been demonstrated to negatively regulate the expression of S1PR1 (Shiow et al, Nature, 2006, Bankovitch et al, J Biol Chem, 2010), so it follows that the receptor expression may be altered in the context of infection if CD69 expression is perturbed.

Taking all of this into consideration, we believe that the expression and function of S1P receptors in the context of HIV infection remains an understudied area of research. We have moderately expanded the background section in our manuscript to reflect the information described here (especially regarding expression of S1PR on CD4 T cells and how this may be altered in the context of infection) and we look forward to continuing to investigate this phenomenon in future work.

2) In figure S1, it would be informative to show a graph with the viability for all donors and doses, especially the highest tested (100 nM).

We agree that it is important to show the viability for all donors and doses, rather than an example donor and time point. We have therefore expanded upon **Supplemental Figure 1** to include viability data for all donors and all concentrations (30-100nM) from the cell-free infection experiments in both NL4-3 infected and NL-AD8-infected donors (**S1 Fig B**) in addition to the flow stain of an example donor, which is now from 72 hours (our longest incubation time, **S1 Fig A**). Moreover, we have added the viability data for all donors for the cell-to-cell transmission experiments which were performed with the second-highest concentration of FTY720 in 7 donors (**S1 Fig C**).

3) Figure 1 panel H should be placed before E, F and G as it is mentioned before in the text and concerns cell-free infection (which should be indicated in the legend).

We have now re-ordered Figure 1 such that it reflects the order that the sub-figures are mentioned in the text. All cell-free infection results are now presented in the same line of Figure 1, followed by the cell-to-cell results below; we hope that this improves the overall clarity.

4) Figure 1 panel A schematic can be confusing and lead to the misleading interpretation that cells used for the cell-to-cell assay were used for the cell-free assay and treated twice with FTY720. Maybe splitting the schematic into two (one for cell-to-cell and one for cell-free) would be clearer. Moreover, as HIV-1 reactivation is assessed later in the manuscript, the experimental design could be rather added as a schematic in figure 2.

We are grateful for this helpful suggestion, which we agree will clarify the manner in which cell-free, cell-to-cell, and latency experiments were performed. We have made the following modifications, as suggested: 1) The schematic for Figure 1 is now split into two schematics, which reflect cell-free and cell-to-cell infection, respectively. This is reflected in the legend. 2) We have added a schematic detailing the experiments using our primary cell model of latency to Figure 2. This schematic is labeled with the time points at which CD4+ (latently infected cells) were isolated and total and integrated HIV DNA were quantified (day 17), as well as the 48 hours of reactivation with Latency Reversal Agents (LRAs, days 17-9) and the assessment of p24-gag by flow cytometry at day 19.

5) For the cell-to-cell results of Figure 1E-G and Figure S2, showing the Gag/CD4 staining in the unlabeled producer cells would help strengthening the conclusion that FTY720 is mainly acting on target cells susceptibility to infection.

We thank the reviewer for this suggestion. We have updated Figure S2 (now **Supplemental Figure 4** (**S4 Fig**) to include the Gag/CD4 staining in the unlabeled (Cell Trace-) producer cells. As can be observed from the figure, the Cell Trace+ (target cells) untreated/ FTY720 treated %p24+ populations were 25.5 and 8.65%, vs 30.8 and 27.6% in the unlabeled (Cell Trace-) producer cells in the representative donor shown.

Moreover, to study the effect of FTY720 on producer cells, infected cells could be cultivated in presence of ART +/- FTY720 and viral production in the supernatant assessed by a p24 ELISA.

Upon the suggestion of the reviewer, we performed this experiment, culturing infected cells from our primary cell model of HIV in the presence of ART (1μM Raltegravir/ 0.5μM Nelfinavir) +/-66nM FTY720 for three days (infected at day 7 and cultured +ART+/-FTY720 days 10-13) and quantified viral production in the supernatant (SN) by p24 ELISA. As can be seen in **Supplemental Figure 5** (**S5 Fig**), we did not observe a significant difference in p24 in the SN of infected cells treated with FTY720 vs untreated, indicating that FTY720 likely does not affect viral release in our model. We believe this data is important as it supports our hypothesis that FTY720 acts mainly by inhibiting that susceptibility of target cells to infection; thus, we have also modified the manuscript to include mention of this data.

Are the effects on cell-to-cell transmission also independent of viral tropism?

This is a great question. In order to determine whether the effect on cell-to-cell transmission is independent of viral tropism, we have repeated the cell-to-cell transmission experiments in several donors infected with R5-tropic virus (NL-AD8) rather than X4-tropic, and observed, overall, a similar effect on inhibition of cell-to-cell transmission as assessed by p24-gag at day 13 in our model (presented in **Supplemental Figure 3** (**S3 Fig**)).

6) How many donors are the results presented in Figure S2 representative of? This should be added in the legend.

We performed labeling with Cell Trace on two donors, and this is now reflected in the legend. Please note that this data is now presented as **Supplemental Figure 4** (**S4 Fig**) due to the addition of supplemental material that is mentioned earlier in the manuscript.

7) The results presented in Figure 2 are somewhat expected. Indeed, it was shown in Figure 1 that the levels of infection at day 13 are lower when cells are treated with FTY720 during crowding. Thus, it is expected that after 4 days of ART, fewer cells will harbor HIV-1 DNA. Adding the compound during the ART treatment might help examining whether FTY720 modulates the seeding of the reservoir per se by examining the proportion of infected cells that will become latently infected.

This is a great suggestion. We have performed this experiment, which we believe has shed interesting light on the data. As can be seen in **Supplemental Figure 6** (**S6 Fig**), we repeated the experiments with our model of latency (as in **Figure 2**), with the modification of treating with 66nM FTY720 during the 4 days of ART treatment (days 13-17, 1μM Raltegravir/ 0.5μM Nelfinavir) rather than during crowding, in order to examine the effect of the compound on seeding of the latent reservoir when inhibiting additional rounds of productive infection. We observed no significant difference in virus reactivated from latency in ART+FTY720 vs ART-FTY720 treatments in donor-matched samples (**S6 Fig B** and **C**). These results indicate that FTY720 may not have a strong effect on modulating the seeding of the latent reservoir in this context, but rather acts mainly on inhibition of initial infection via the various modes presented in the manuscript. In addition, we assayed the viral titer by p24 ELISA following three days of ART+/-FTY720 (**S5 Fig**). Please see the detailed description of this experiment above. We would like to thank the reviewer for this suggested experiment, which has added depth to the manuscript.

8) In panels A-D of figure 2, it seems surprising to reach p values of <0.0001 with a Wilcoxon test on 5 donors.

We apologize that we made a mistake with indicating the statistical test used on these data sets. Rather than a Wilcoxon test, the total and integrated HIV DNA PCR results were analyzed by Paired T test. We have indicated the correct test in the figure legend.

9) In panels A-D of figure 2, indicating whether we are looking at total or integrated DNA would make the figure clearer.

We apologize for the confusion. We have added titles to these four sub-figures to indicate whether total or integrated DNA is being displayed.

10) Do they authors have any data or hypotheses for the mechanisms of downregulation of CD4 presented in Figure 3. This could be at least discussed.

The downregulation of CD4 shown in **Figure 3** was indeed an interesting result. We have expanded the section of the discussion pertaining to the reduced MFI of CD4 on CD4 T cells treated with FTY720 to mention potential mechanisms for this downregulation. While the downregulation of CCR5 was less surprising due to its reported coexpression with S1PR1 (Duquenne et al, AIDS, 2017), to our knowledge, there are no reports of CD4 colocalization with S1P receptors on CD4 lymphocytes, although, as previously mentioned, S1PR1 and S1PR4 are known to be expressed on CD4 T cells and respond to FTY720. We hypothesize that this decrease in CD4 abundance may be related to the less activated phenotype of CD4 T cells cultured with FTY720 (**Figure 4**) or potentially a disturbance in the integrity of the membrane that may correspond to an internalization/ downregulation of CD4. Please also see the expanded discussion section.

11) In figure 4, why did the authors wait until day 10 for treatment with FTY720?

In the cell cycle profiling experiments presented in this figure, we decided to analyze the effect of FTY720 on cell cycle at this time point to mimic as closely as possible the effect observed in the cell-to-cell transmission experiments, which were performed at day 10 of our model of HIV infection (see **Figure 1** right schematic and **Fig. 1 F-H**). We did not want to over-state the effect that the compound may have on cell cycle in our model, as the phenotype of cells from approximately day 7 onward (cell-to-cell and latency experiments) is more representative of a “Cultured T Central Memory cell (T_CM_)” phenotype than cells from earlier in the model (see Bosque and Planelles, Methods, 2011 and Bosque at al, Blood, 2009); thus, we elected to examine the effect of FTY720 at day 10. We realize that this may not have been clearly stated initially, and we have now clarified this in the text pertaining to **Figure 4**.

12) In figure 4 panels B, C and D, adding a legend (grey, green) on the figure would improve the readability and performing statistical analysis would strengthen the interpretation.

We appreciate this suggestion. We have added a legend (grey= untreated, green= FTY720) and have performed statistical analysis, which is now reflected in the legend. The reduction in S/G2 cell cycle phase with FTY720 treatment is statistically significant at 48 and 72 hours (p= 0.028 and 0.043 for 48 and 72 hours, respectively) by Multiple T Test. This is now indicated by asterisks on the figure and explained in the legend. The differences observed for G0/ G1b phase of cell cycle are not statistically significant.

13) Relative to Figure 4, in the context of infection, does treatment with FTY720 impact the cell cycle or proliferation of infected cells?

We would like to thank the reviewer for the interesting question. In order to determine whether treatment with FTY720 impacted proliferation of infected cells, we infected cells from our model with NL4-3 at day 7 and treated +/- 66nM FTY720 from day 10-13 during crowding (as done in **Figure 1** and **S3 Fig**), then stained for Ki67 expression at day 13. While the number of donors we have been able to assess thus far has been limited to two infected donors, in these donors FTY720 had a clear effect on reducing the expression of Ki67 both in uninfected and HIV-infected cells. This data is presented in **Supplemental Figure 7** (**S7 Fig**) and has raised the interesting question of how FTY720 treatment may impact proliferation of the infected pool of cells during HIV infection. This trial experiment has prompted us to look deeper into the effects of treating with FTY720 in the context of proliferating, infected cells in the future.

14) To really look at the importance of SAMHD1 in the effects of FTY720, the authors could repeat the experiments in cells treated with VLP-Vpx. Looking at whether HIV-2 replication, which counteracts SAMHD1, is less inhibited by FTY720 could be of interest in a future study.

We are indeed very interested in further pursuing the mechanism of the potential effect of FTY720 on SAMHD1, and we thank the reviewer for this helpful suggestion. This is an ongoing area of investigation in our lab and is tentatively planned to be included in a future manuscript. For this study, our objective was to offer an overview of the potential of an FDA-approved drug to target multiple stages of the HIV-1 life cycle; thus, a deeper dive into the mechanism of SAMHD1 regulation is out of the scope of this manuscript, but is certainly of interest for the future.

Upon the suggestions of the reviewer, we repeated the cell-free infection experiments in primary CD4 T cells treated with VLP-Vpx and were able to obtain preliminary data in our model. As can be seen below, in the donors examined, VLP-Vpx had a modest overall effect in increasing frequency of infected cells (%p24+) when CD4 T cells were treated at day 6 of our model and infected from days 7-10 (**A**, **B** and **C**); however, we did not observe a notable decrease in total SAMHD1 levels 24 hours post-VLP-Vpx spin infection by Western blot (**D**). These results may be in keeping with previous work by Descours et al (Retrovirology, 2012) who reported that VLP-Vpx treatment of quiescent CD4 T cells increased permissiveness to HIV infection but demonstrated that VLP-Vpx-mediated degradation of SAMHD1 appeared much less efficient in resting CD4 T cells than in myeloid cells. This group did not report a decrease in SAMHD1 in CD4 T cells by Western blot and showed only a moderate decrease in SAMHD1 levels by flow cytometry 4 days following VLP-Vpx treatment. A strong decrease in SAMHD1 levels was not observed unless CD4 T cells were activated and stably transduced to express Vpx. Moreover, this group used a reporter virus with GFP under control of a CMV promoter, a system which differs greatly from ours, in which we infect primary cells with an NL4-3 molecular clone. In seminal work by Baldauf et al (Nature Med, 2012), infection of CD4 T cells with an HIV-1 construct expressing Vpx resulted in increased permissiveness to infection and degradation of SAMHD1. Considering that we and others did not observe a notable decrease in SAMHD1 by Western blot in unstimulated resting cells, employing HIV-2, as suggested by the reviewer, may be better suited to investigation of the role of SAMHD1 in our primary cell model, and we are eager to employ these methods in future work.

As an alternative approach to probe the involvement of SAMHD1 in FTY720-induced decrease in infection in our model, we investigated the potential increase in activity of SAMHD1 by RT-PCR-based dNTP quantification. Our preliminary results indicate that FTY720 may indeed increase the activity of SAMHD1 resulting in a decrease in dNTP levels. Please see the detailed response to Reviewer 3 (question 5) below for a full description of the dNTP experiment and the preliminary data.

15) Panel 5D is redundant with 5B and 5C.

Thank you for this point. We agree that Panel 5D is somewhat redundant with the previous panels, and we have removed this sub-figure.

16) Panel 6G title should be Cyclin D2 (space missing) and 6H should be p21 (not p27).

Thank you; we have corrected the Cyclin D2 and p21 graph titles.

17) Results presented in Figure 7 suggest that FTY720 is rather inactive for latency reversal. Repeating these experiments in ART-treated patients’ samples would strengthen the conclusions on the role of FTY720 during latency reversal. Moreover, how may FTY720 modulate CD4 T cell activation? This could be discussed.

We would like to thank the reviewer for this comment. We agree that FTY720, on its own, has very weak LRA activity (comparable to that of IL-2 in our model, which serves as a very weak LRA control). In fact, we did not anticipate that FTY720 would have LRA activity in our model. Others have reported that FTY720 and a specific S1PR1 antagonist could both reactivate latent HIV in primary cells and showed in reporter cells that LTR activation appeared to occur in an NFκB-dependent manner (Duquenne et al, AIDS, 2017), but the transcription factors necessary for HIV reactivation in our model (Bosque and Planelles, Methods, 2011) likely differ, as do the source of primary cells and the model of HIV latency used. Therefore, we were mainly interested in verifying that FTY720 would not interfere with the activity of several established LRAs (**Figure 7**). We apologize if we were at all unclear describing this figure. The suggestion to examine the role of FTY720 during latency reversal in ART-treated patient samples is very interesting, and we would be eager to perform this experiment in the future.

In order to probe how FTY720 may modulate T cell activation in the context of latency reversal, we performed an additional trial experiment. We reactivated latently infected T_CM_ from day 17 (as in **Figure 2A** and **Fig 7**) of our model in the presence or absence of 66nM FTY720 and analyzed activation marker (CD69, CD25) expression by flow cytometry. We observed a moderate decrease in the expression of these activation markers when cells were activated in the presence of FTY720 (preliminary data shown below), although we had previously observed that there was no effect of FTY720 on reactivation of latent HIV by various LRAs (**Figure 7**). Interestingly, this may be in agreement with reports that FTY720 may inhibit both TCR-dependent and independent activation in primary human T cells, although these experiments were not performed in T_CM_ as our studies were (Baer at al, Sci Reports, 2018).

On the other hand, when T_CM_ were co-cultured with ART+/- 66nM FTY720 from day 13-17 followed by CD4+ isolation on day 17 and 48 hours of reactivation, we did not observe a notable difference in expression of the activation markers CD69 or CD25 at day 19 (data shown below, 4 donors total for CD69 and 3 for CD25). Moreover, when T_CM_ were co-cultured with ART+/- 66nM FTY720 from day 13-17, there was no effect on reactivation of latent virus at day 19 (see **S6 Fig**). Altogether, this indicates that FTY720 does not appear to greatly alter the activation of T_CM_ from our model at multiple time points, although the expression of CD69 is decreased in some donors with FTY720 treatment. Yet, this phenotypic alteration does not correspond to an impact on reactivation from latency in our model by various LRAs. This question is deserving of further investigation in the future.

18) In the discussion, line 323, it is stated that there is an “increased expression of active SAMHD1”. This should be changed to “relative expression” as what was observed is a lower expression of the inactive phosphorylated form.

Thank you for pointing this out. The line now reads, “increased relative expression of the active form of SAMHD1.”

19) In the Methods section, line 409 should read “Results were expressed as HIV DNA copies per 106 CD3 T cells”.

We have corrected this statement to read “CD3 T cells.”

**Reviewer #3:** [**Note:** Comments copied from Summary section and addressed below.]

1) There is a general lack of important details with many of the figures. For example, in all figures with scatter plots, it is not really clear what is plotted. Mean + SD? Mean + range? How is the data normalized when relative percent are shown? This is especially important for the western blot figures 5 and 6. Is the data standardized to the loading control as it should be?

We would like to apologize for the lack of clarity in the figures. Firstly, we have indicated more precisely the information plotted. In all instances of scatter plots, the Mean + Standard Deviation (SD) is plotted. We have now indicated this in the figure legends. We have also indicated in the figure legends the equations used to normalize data when showing relative infection. For the relative productive infection, we determined relative infection as: **% relative infection= ((d13_treat_-d10_treat_)/(d13_untr_-d10_untr_))*100** and for relative latent infection, we used the equation: **% relative infection= ((d19_treat_-d17_treat_)/(d19_untr_-d17_untr_))*100.** These equations are now clearly listed in the respective figure legends.

We would like to thank the reviewer for also pointing out the need for clarity in the Western blot figures. For all Western blots, normalization was performed by standardizing the data of each treatment to the respective β-actin loading control. This is now indicated in the figure legends of Figs 5 and 6. Moreover, we have presented the entirety of the Western blots and loading controls in **Supplemental Figure 8** (**S8 Fig**).

2) The authors stated that "FTY720 did not alter viability at any of the concentrations tested" (line 129), but figure S1 only shows one concentration (66nM). Viability should be shown for all the concentrations.

As detailed in the response to Reviewer 2 above, we have expanded **Supplemental Figure 1** to include viability data for all concentrations (30-100nM) tested in cell-free infection experiments (7 donors each for NL4-3 and NL-AD8). Additionally, we have included a compilation of the viability of all donors tested in cell-to-cell transmission experiments (N=7).

3) An important control for figure 2 should be an inclusion of viability data following treatment for 3 days with FTY720 to show that the reduction in HIV DNA is not due to cell death. Viability data in Figure S1 is from 48hrs.

This is a very good point. As described above, we have now included the viability data from all donors treated for three days with FTY720 (N=7). The example flow plots are also now from 72 hours rather than 48.

4) Line 236, “we observed a significant reduction in pSAMHD1 (39.63 +/- 14.47%, Figs 5A and 5B) but did not observe an effect on total SAMHD1 levels (Figs 5C and 5D)”. Can the authors comment on the fact that it appears that there are changes in total SAMHD1 levels (figure 5C), but perhaps inconsistent across the donors?

Thank you for this observation; we indeed observed donor variability in the effect of FTY720 on total levels of SAMHD1 in the six donors we examined. In two of the donors assayed, we observed an increase in total SAMHD1 levels, whereas in the other four donors, total SAMHD1 levels decreased slightly. We examined the possibility that this effect could be age or biological sex dependent (activity of SAMHD1 in primary human macrophages has been shown to be dependent on biological sex, with those cells derived from female donors being less susceptible to infection with HIV-1 (Szaniawski, Spivak, Bosque and Planelles, J Infect Dis, 2019)). However, these variables do not appear to significantly affect the alteration of SAMHD1 in our model, at least among the donors we have assayed thus far. As there is already donor variability in the constitutive expression of this protein (**Figure 5**) as well as in cell cycle status (**Figure 4**), perhaps the response to FTY720 may vary by donor as well. Due to this inherent variability, we elected to simply show the relative change in SAMHD1 with treatment (expressed as percentage of expression of untreated) and overall there was no significant change. It would be interesting in the future to further examine whether biological sex or age may influence the phosphorylation or activity of SAMHD1 in our model.

5) Line 247, the authors state that that their results “results suggest a model of regulation of CDK4/6 by Cyclin D3, which in turn results in a decrease in phosphorylation and corresponding activation of the activity of the restriction factor SAMHD1.” However, they did not really provide direct evidence of SAMHD1 activation. A functional assay showing SAMHD1 activation would be necessary to support their claim.

We would like to thank the reviewer for pointing out that our wording may not entirely reflect our observations, and we apologize for inadvertently overreaching with our conclusions. We have modified the text of the manuscript to read “Our results indicate a relative increase in the active (non-phosphorylated) form of SAMHD1, which may be associated with the decrease we observed in Cyclin D3. The mechanism of the regulation of SAMHD1 in our primary cell model remains to be elucidated.” We hope that this change to the text clarifies that we are reporting an observed association and not a direct mechanism. As detailed in the response to Reviewer 2 above (14) an in-depth examination of the regulation of SAMHD1 in our model is out of the scope of this manuscript, but is the subject of ongoing investigation.

We do have preliminary data quantifying dNTP pools in cultured T_CM_ from our primary cell model which indicates that FTY720 treatment does increase the activity of SAMHD1 resulting in a decrease in cellular dNTP pools. We have worked to optimize the RT-PCR-based dNTP quantification assay published by Wilson et al (Nucleic Acids Res., 2011) for detection of nucleotide pools in our Cultured T_CM_. As can be seen from the preliminary data below, T_CM_ from our model expressed very low picomolar levels of dNTPs and FTY720 treatment (66nM) for 24 hours decreased dNTP pools (shown are duplicate data points from three individual donors, with the nucleotide assayed indicated. Symbols indicate the nucleotide assayed: triangles= cytosine, diamonds= guanine, stars= thymine). However, this assay requires further optimization for use in cells from our model, due to the very low levels of dNTP pools in T_CM_ and the fact that levels of each individual nucleotide varies across donors.

In light of this, we agree with the reviewer and have amended our statement to reflect that we are reporting an association of relative non-phosphorylated SAMHD1 levels with FTY720 treatment. We are very interested in continuing to optimize this experimental technique for detection of low levels of dNTPs in the future.

6) Given that FTY720 is an immunomodulatory agent, and their data shows an effect on the cell cycle, including an increase towards a more resting, G0 phenotype following FTY720 treatment, can the authors comment on 1) the status of activation of their Tcm and how that might influence some of their results, including a limit in HIV infection and 2) the level of transcription factors necessary for efficient HIV transcription (e.g . P-TEFb) in cells treated with FTY720 and how that might also affect their observations on HIV infection?

For many of the experiments presented in this manuscript, primary cells were cultured for up to 10 days before FTY720 treatment and infection. Therefore, these cells represent a “Cultured T_CM_” phenotype (Bosque and Planelles, Methods 2011). Generally, their phenotype pertains to that of a semi-resting but transcriptionally active T_CM_ and as such they are susceptible to infection with HIV but are not generally infected as readily as more activated CD4 T cells (i.e. cells from day 5 of our model). In keeping with this, we observed that the overwhelming majority of T_CM_ from our model (cultured until day 10) pertained to the G1b stage of the cell cycle when we examined cell cycle stage by RNA/DNA staining (**Figure 4**). There was, unsurprisingly, moderate donor variability with respect to this. For these reasons, it is not surprising that we do not routinely observe high levels of infection in these cultured primary cells.

In order to probe how FTY720 may influence activation state of T_CM_ we examined markers of activation by flow cytometry on T_CM_ from days 10-12 of our model, either treated or not treated with FTY720, and observed that CD69 and CD25 are moderately expressed on these cells but not significantly altered by FTY720 treatment (shown below; 4 donors total).

We have previously demonstrated that in T_CM_ from our primary cell model of HIV latency, various transcription factors are required for efficient HIV-1 transcription depending on the reactivation stimulus used (Bosque et al, Blood 2009). Interestingly, Duquenne et al (AIDS, 2017) observed that stimulation of S1PR1 with FTY720 or an S1PR1 antagonist reactivated HIV from latency, apparently in an NFκB- dependent manner. In our model, which differs from the Duquenne model both with regard to setup of the model and the phenotype of the cells used, FTY720 did not impact the reactivation of HIV with any of the latency reversal agents (LRAs) employed, including SAHA, Ingenol, or CD3/28 stimulation, among others (**Figure 7**). This suggests that modulation of S1P signaling with FTY720 does not impact transcription factors required for efficient HIV-1 transcription in our model.

**Reviewer #3:** [**Note:** Comments copied from Major Issues section and addressed below.]

1) An important control for figure 2 should be an inclusion of viability data following treatment for 3 days with FTY720 to show that the reduction in HIV DNA is not due to cell death. Viability data in figure S1 is from 48hrs.

Thank you for pointing this out. Please see our response to (3) above. We have now included viability data from all donors treated with FTY720 for 72 hours in **Supplemental Figure 1**.

2) Viability data for all concentrations of FTY720 should be included.

Please see our response to (2) above. We have added viability data for all concentrations of FTY720 tested (30-100nM) to **Supplemental Figure 1**.

3) The authors need to provide direct evidence to support their claim that FTY720 treatment results in an increase of activated SAMHD-1.

This point is well-taken. We would like to apologize if we have overreached with our statement that FTY720 treatment results in an increase in activated SAMHD1. More accurately, we observed a relative decrease in inactive (phosphorylated) SAMHD1, which suggests a possible increase in activity. While we are very interested in further pursing the detailed mechanism of regulation of this innate restriction factor in our model and this is indeed an ongoing area of investigation, with this manuscript we aimed to provide a first look at the modulatory effect of FTY720 on multiple steps of the HIV-1 life cycle.

We have performed additional experiments to address the function of SAMHD1 including a VLP-Vpx assay and dNTP analysis as described above. Our preliminary dNTP quantification data indicates that treatment with FTY720 appears to increase the activity of SAMHD1 as measured by decreased dNTP pools, although these experiments require further optimization for use with resting cells. In agreement with the reviewer, we have modified our text to more accurately reflect the association rather than causation observed between FTY720, Cyclin D3, and SAMHD1. The detailed mechanism of our proposed cell-cycle-dependent regulation of SAMHD1 activity induced by S1P signaling modulators is an ongoing area of investigation in our laboratory and is deserving of additional consideration in an upcoming manuscript.

[Please also see our detailed response to the comment above (5) as well as the response to Reviewer 2 (14) above.]

Part III – Minor Issues: Editorial and Data Presentation Modifications


**Reviewer #1:**

The viral titer should be quantified in some experiments (eg: Fig. 1) to show if the treatments affect virus production.

We are thankful for this suggestion, and the quantification of viral titer is indeed a useful addition to determine whether FTY720 affects production of virus, which is important to address. As detailed above in our response to Reviewer # 2, we have quantified p24 by ELISA following three days of FTY720 treatment in the presence of ART in order to determine the effect of the treatment on viral production. As can be seen in **Supplemental Figure 5**, there was no noticeable difference in viral titer with FTY720 treatment at either of the concentrations tested (66nM and 100nM). This supports a role of FTY720 in reducing susceptibility of target cells to infection rather than in altering viral production.

In Fig. 2: absolute number of copies should be presented.

We apologize that this figure was unclear. We have now labeled all sub-figures so that it may be more easily seen whether absolute number or relative copies are being presented (both absolute copies and relative are shown for total and integrated HIV-1 DNA in **Figure 2 B-E**).

In Fig. 3D: there is no visible change on the BLAM assay for at least donor 1.

This is true; in our primary cell model of HIV we routinely see donor-dependent variability. In fact, the donor with no visible change was one of the last donors assayed, and the increase in the N of the experiment actually resulted in a more significant result overall, in spite of the variability. Due to the spread of results commonly observed in primary cells, we have ensured that to the best of our ability we included as robust a sample size as possible in all experiments.

In Fig. 4: show cell cycle profile.

We apologize for any unclarity in the presentation of Figure 4. This assay was a cell cycle assay in which we stained RNA with Pyronin Y and DNA with 7-AAD and expressed the following populations graphically: **G0**, **G1b**, and **S/G2** (see also Toba et al, J Immunol Methods, 1995 and Korin and Zack, J Virol, 1998). We have corrected the plot to reflect these populations and have clearly labeled the populations on each plot in **Figure 4**. Moreover, cell cycle histograms from an example donor are presented below. Due to the large proportion of our Cultured T_CM_ cells corresponding to the G1b phase of cell cycle, we found that expression of flow cytometry dot plots to determine these populations proved much more precise and sensitive than applying cell cycle modeling in FlowJo software to generate histograms.

**Reviewer #2:** (No Response)

**Reviewer #3:**

1) Minor, but confusing, Figure 1 in text not in sequence. 1B, 1C, 1D then 1H. Should be fixed.

We have reordered Figure 1 to reflect the order of figures mentioned in the text and hope that the figure is now clearer. Please see also response to Reviewer #2 above.

2) In figures with scatter, it would make it easier for the reader to evaluate the response across donors if a unique symbol is used for each donor.

We appreciate this point. In several sub-figures in which it would be beneficial, we have connected the data points (dots) rather than show scatter to help the reader evaluate the response of each donor (i.e. **Figs 1G**, **Fig. 4F** and **S3 C**). For other figures with scatter, we have now represented each donor with a unique symbol (**Fig. 2G**, **Fig.** **3 E-G**, and **Fig 7**.)

3) As Western blot densitometry tracing is only semi-quantitative, the blots for all the donors should be included as a supplementary figure to better help the reader evaluate the data.

We agree completely with this suggestion and have now presented the entirety of the Western Blot data (N=6 for all blots) as well as the corresponding β-actin blot for each respective protein in **Supplemental Figure 8**, in order to assist with evaluation of the data.
