# Supplementary material for: Fingolimod inhibits multiple stages of the HIV-1 life cycle
Source: PLoS Pathog. 2020 Aug 13;16(8):e1008679. doi: 10.1371/journal.ppat.1008679 (PMC7425850; doi:10.1371/journal.ppat.1008679)
Supplement: Attachment — Submitted filename: Response to Reviewer Resop et al 06.01.20.docx [file ppat.1008679.s010.docx]

We would like to thank the editor and reviewers for the thorough review of our manuscript. We are happy to have made the minor revisions requested, which we believe have strengthened the manuscript. Please see our detailed responses to the reviewer below (in green).

Reviewer #2:

1) In Figure S2B and S3C, include statistics. In Figure S5B and S6C legend, the statistical test performed should be indicated. Indeed, in most of the manuscript, Wilcoxon tests are used, but with 4 donors here, it is not possible to use such a test.

We thank the reviewer for this suggestion and apologize for the omission of statistical analysis in these supplemental figures. We have now added statistics to Figures S2B and S3C and the test used (paired t-test in both cases) is indicated in each figure legend. We also appreciate the reviewer pointing out that a Wilcoxon test should not be utilized for analysis of 4 donors. Thus, for Figures S5B and S6C, we have made a minor correction in the test used and have indicated in the figure legends that a paired t-test was performed for each data set.

2) Although the new Fig S7 is appreciated, an overlay of 4 histograms is difficult to read. Maybe presenting an offset histogram or plotting the Ki67+ percentages would make this figure clearer.

We appreciate this suggestion. The Ki67+ populations in Fig S7 are now presented as an offset histogram with percentages of Ki67 shown. We believe this improves the clarity of the figure.

3) In the abstract: “FTY720 reduces establishment of latent HIV infection” might be confusing given the new Fig S6 that shows that FTY720 does not impair the seeding of the viral reservoir per se. This could be rephrased to avoid any confusion.

We agree with the reviewer that rephrasing this part of the abstract is appropriate in order to avoid confusion. The abstract now reads “We show that FTY720 (Fingolimod), an FDA-approved functional antagonist of S1P receptors, blocks cell-free and cell-to-cell transmission of HIV and consequently reduces detectable latent virus.” We believe this more accurately depicts the significance of our observations.

4) The description of Fig 2 is sometimes misleading. Indeed, the reduction in integrated HIV DNA at day 17 and the decrease of p24+ cells upon reactivation at day 19 may be the consequence of the lower levels of productive infection observed at day 10 and 13. Concluding that FTY720 leads to “a reduction of the seeding of the latent reservoir in primary CD4 T cells” may be an over statement, given the results of Fig S6. The authors should discuss that the reduction is likely a consequence of a lower productive infection rather than seeding of the reservoir.

This point is well-taken, and we have now modified the description of Figure 2 accordingly. The pertinent section of the description of Figure 2 now reads:

“In light of our previous work demonstrating that in this model latently infected cells are established during cell-to-cell transmission, these results indicate that the brunt of the role of FTY720 on reduction of latency is likely a consequence of the reduction in productive infection. Overall, our results indicate that FTY720 not only reduces productive infection but that the effect is carried over into a reduced incidence of latently infected primary CD4 T cells.”

5) In the description of Fig S6 in the text, the authors may mention that the results indicate that in this model, latency is established during ART but is not impacted by FTY720.

We would like to thank the reviewer for this suggestion. We have reworded the description of Figure S6 to read:

“Interestingly, when FTY720 was added along with ART from days 13-17, there was no significant difference in virus reactivated from latency at day 19, indicating that in this model latent infection is established prior to ART and following ART exposure the latent reservoir is not impacted by FTY720 (**S6 Fig**).

6) Similarly, in the discussion “Therefore, our results suggest that FTY720 may be useful as a strategy to limit the size of the latent reservoir if used concomitantly with ART initiation”. This hypothesis is not supported by the novel Fig S6.

This is a very good point and we are grateful to the reviewer for bringing this to our attention. We have removed this statement from the discussion. The discussion now reads:

“Recently, Abrahams *et al* demonstrated that most latently infected cells are generated near the time of ART initiation [81]. When we treated cells in our model with FTY720 concomitantly with ART, we readily observed reactivation of HIV, albeit no difference in FTY720-treated or untreated samples. Therefore, results in our model suggest that FTY720 may be useful as a strategy to limit the size of the latent reservoir if used prior to ART initiation, such as in acute infection.”

7) In Fig 3, presenting examples of fusion plots before the graph with all the donors would facilitate reading. Fig 3B and 3C are somewhat redundant.

We agree that presenting the examples of the fusion plots before the graph with all donors is helpful to the reader, and we have reorganized Figure 3 accordingly. We also agree that the sub-figures showing absolute fusion events and relative fusion are somewhat redundant, but to retain the overall readability of the figure and to present the data in different ways (as human donors showed variability for fusion events), we have retained both Figures 3 B and C.

8) In Fig 5A and 6A, blot images should be surrounded with a black line to make cuts clearer. Please assign the molecular weights to the corresponding panel. Molecular weights should be added in Fig 6A and S8.

We agree that these modifications increase the clarity of Figures 5A, 6A, and S8, and we have now made these changes; moreover, we have added molecular weights to all three panels (Fig 5A, Fig 6A, and Fig S8).

9) The authors should discuss the differences with the study of Duquenne C et al. (2017) where it was shown that S1PR1 agonists reverse latency, contrary to the results presented here.

We thank the reviewer for pointing out that discussion of the results of Duquenne et al would add to the manuscript. The *in vitro* system used by this group to examine viral reactivation differed in several ways from our model, including the cell type (resting PBMCs vs our cultured T Central Memory cells), viral tropism (R5 vs X4, respectively) and the S1PR1 antagonist/ agonist used (Duquenne et al utilized SEW2871, a specific S1PR1 agonist, in addition to FTY720). Indeed, when examining p24 production following stimulation with the various antagonists/agonists, a significant difference from the negative control was reported only with S1P itself and SEW2871, not FTY720. It is possible that some or all of these factors contributed to the different results our groups observed. We have modified the discussion section accordingly, and it now reads:

“Finally, we examined whether FTY720 would interfere with the ability of a panel of established LRAs to reactivate latent HIV in our model. A previous report by Duquenne *et al* indicated that an S1PR1 agonist, SEW2871, could induce viral production in an *in vitro* model of HIV latency in resting PBMCs [14]; however, in our primary cell model of latent HIV infection, we observed only weak LRA activity of FTY720 following 48 hours of culture, likely due to differences in the models used as well as differing mechanisms of FTY720 and other S1PR1 antagonists/ agonists. Importantly, we did not observe an impairment by FTY720 of the activity of several well-established LRAs, indicating that FTY720 may be used in combination with other LRAs towards reducing the latent HIV reservoir.”

Again, we are grateful to the editor and reviewers for the comprehensive review of our manuscript and thank you for your time.
